# Comparison of Biomechanical Performance of Five Different Treatment Approaches for Fixing Posterior Pelvic Ring Injury

**DOI:** 10.1155/2020/5379593

**Published:** 2020-01-22

**Authors:** Yongtao Lu, Yiqian He, Weiteng Li, Zhuoyue Yang, Ruifei Peng, Li Yu

**Affiliations:** ^1^Department of Engineering Mechanics, Dalian University of Technology, Dalian 116024, China; ^2^State Key Laboratory of Structural Analysis for Industrial Equipment, Dalian University of Technology, Dalian 116024, China; ^3^DUT-BSU Joint Institute, Dalian University of Technology, Dalian 116024, China; ^4^Department of Orthopedics, The First Affiliated Hospital of Dalian Medical University, Dalian, China

## Abstract

**Background:**

A large number of pelvic injuries are seriously unstable, with mortality rates reaching 19%. Approximately 60% of pelvic injuries are related to the posterior pelvic ring. However, the selection of a fixation method for a posterior pelvic ring injury remains a challenging problem for orthopedic surgeons. The aim of the present study is to investigate the biomechanical performance of five different fixation approaches for posterior pelvic ring injury and thus provide guidance on the choice of treatment approach in a clinical setting.

**Methods:**

A finite element (FE) model, including the L3-L5 lumbar vertebrae, sacrum, and full pelvis, was created from CT images of a healthy adult. Tile B and Tile C types of pelvic fractures were created in the model. Five different fixation methods for fixing the posterior ring injury (PRI) were simulated: TA1 (conservative treatment), TA2 (S1 screw fixation), TA3 (S1 + S2 screw fixation), TA4 (plate fixation), and TA5 (modified triangular osteosynthesis). Based on the fixation status (fixed or nonfixed) of the anterior ring and the fixation method for PRI, 20 different FE models were created. An upright standing loading scenario was simulated, and the resultant displacements at the sacroiliac joint were compared between different models.

**Results:**

When TA5 was applied, the resultant displacements at the sacroiliac joint were the smallest (1.5 mm, 1.6 mm, 1.6 mm, and 1.7 mm) for all the injury cases. The displacements induced by TA3 and TA2 were similar to those induced by TA5. TA4 led to larger displacements at the sacroiliac joint (2.3 mm, 2.4 mm, 4.8 mm, and 4.9 mm), and TA1 was the worst case (3.1 mm, 3.2 mm, 6.3 mm, and 6.5 mm).

**Conclusions:**

The best internal fixation method for PRI is the triangular osteosynthesis approach (TA5), followed by S1 + S2 screw fixation (TA3), S1 screw fixation (TA2), and plate fixation (TA4).

## 1. Background

Pelvic fractures and injuries account for 10–25% of all fractures in the body and mostly result from high-energy traumas such as traffic accidents and crushing and falling injuries [1]. A high mortality rate approaching 28% is associated with pelvic fractures, which seriously threaten the quality of life of patients [2]. Posterior pelvic ring fractures are relatively difficult to manage because the pelvis has an irregular and complex cortical surface. Common complications associated with surgery for pelvic ring fractures are pelvic deformity, limb shortening, recurrent fracture site, and so on [3]. Therefore, challenges still exist for orthopedic surgeons to find an effective treatment approach for fixing posterior pelvic ring fracture injuries.

Over the past several decades, a variety of fixation techniques have been employed to repair posterior pelvic ring fractures [4–8], including locking compression plates [6], posterior crew fixation [9], pedicle screw-rod fixators [7, 10], plate-screw fixation [11], and transiliac internal fixators [12, 13]. To evaluate the performance of these different techniques, mechanical testing using cadavers and clinical prospective and retrospective studies are commonly used [6, 7, 9, 11–15]. However, in in vitro mechanical testing, a large number of cadavers are always required due to the intersubject variances, and it is a major challenge to simulate different fixation approaches using the same specimen. Additionally, the results from different specimens are influenced by variations in bone quality and anatomy, fracture pattern, and fixation location. Prospective and retrospective studies always require a prolonged period for recruiting and following patients. In addition, intersubject differences and patient lifestyle histories may have a large influence on the outcomes. Therefore, many unsolved issues remain regarding the biomechanical performance of different fixation techniques.

The finite element (FE) modeling technique enables the exploration of different scenarios using computer models, and once validated, it can serve as an effective tool in biomechanics. Therefore, in recent years, the FE technique has been widely used to investigate the biomechanical performance of different techniques [16–22], including analyzing different types of S1 screw fixation [22] and evaluating different fixation techniques for the treatment of sacroiliac joint injuries [18]. However, only a few studies have investigated the biomechanical stability of different fixation techniques for posterior pelvic ring fractures. In particular, no study has evaluated the performance of the modified triangular osteosynthesis (TOS) fixation approach [23]. Compared with standard fixations, the modified triangular osteosynthesis creates additional resistance to vertical displacement and rotation and thus might be more suitable for fixing posterior pelvic ring fractures. Therefore, a comparison study using FE analysis will provide additional important information on the mechanism of fixing pelvic ring fractures that cannot be obtained from clinical and in vitro testing studies (e.g., the stress and strain distribution inside the structure). Additionally, this comparison can provide guidance on the choice of an appropriate fixation approach in the clinical setting.

The aim of this study was to investigate the biomechanical performance of a modified triangular osteosynthesis fixation approach for posterior pelvic ring injury by comparing it with four other fixation approaches using a finite element model of a spine-pelvis complex.

## 2. Methods

### 2.1. Clinical CT Scan

A 29-year-old healthy male with no history of pelvic tumor, bone injury, or deformity was selected for this study. The spine-pelvis complex, including the lumbar vertebrae, sacrum, and pelvis, was scanned using a 64-slice LightSpeed computed tomography (CT) scanner (Phillips, Netherlands). The CT images were acquired using an image plane resolution of 0.69 × 0.69 mm and a slice space of 1.0 mm with a reconstructed image voxel size of 0.69 × 0.69 × 1.0 mm. The CT scan was performed six times over a period of two years (three scans per year) to ensure that the radiation exposure was within acceptable limits for a young male. A slight overlap of the images between CT scans was ensured to facilitate the assembly of images afterwards.

This study protocol was approved by the local Ethics Committee of First Affiliated Hospital of Dalian Medical University (no. YJ-KY-FB-2016-47). Written informed consent was obtained from the participants. All experiments were performed in accordance with relevant guidelines and regulations.

### 2.2. Finite Element Model of the Spine-Pelvis Complex

**Figure 1:**
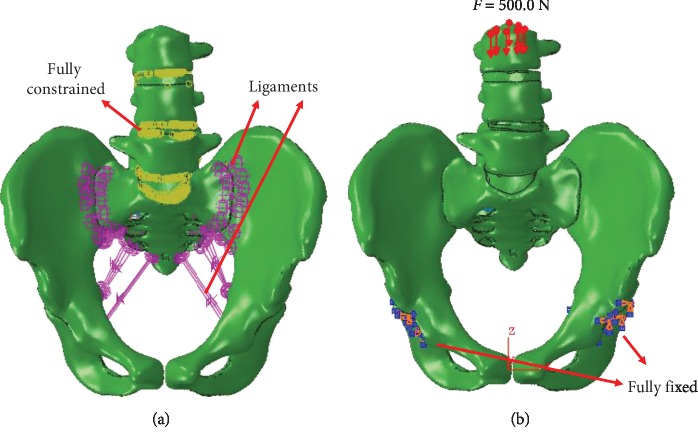
Finite element model of the spine-pelvis complex. (a) Illustration of ligaments and interaction constraints between discs and vertebrae (front view) and (b) illustration of boundary conditions (front view).

The finite element model of the spine-pelvis complex, including the lumbar vertebra (L3–L5), sacrum (S1–S5), and pelvis, was generated from the clinical CT images (Figure 1). First, different bones were segmented using a semiautomatic process based on the image grayscale values using the image processing software Mimics (v16, Software and Services for Biomedical Engineering, Materialise HQ, Belgium). Three-dimensional (3D) geometries of bone components were then smoothed using Geomagic Studio (v12, Raindrop Geomagic, NC, USA). After generating the FE meshes in HyperMesh (Altair Engineering, Troy, MN, USA), the FE model was imported to Abaqus (v6.12, Dassault Systemes Simulia Corp., Providence, RI, USA) for the FE calculations.

In the FE spine-pelvis complex, the bony parts included the vertebra, the sacrum, and the pelvic bone (left and right ilium and pubis), which consisted of the cortical bone, the endplates, the cancellous bone, and the posterior elements. The interfaces between the superior and inferior articular processes and between the sacrum and pelvis were modeled with a frictionless contact. The mechanical behaviors of the cortical shell, the cancellous bone, the endplates, and the posterior elements were simulated using isotropic linear elastic models (Table 1), the material constants of which were selected based on data in the existing literature [24–27]. The thicknesses for the cortical shell and endplates in the FE model were assumed to be 1.3 mm and 0.8 mm, respectively [29, 30]. All the bony parts were meshed using a 4-node tetrahedron element (C3D4).

In the FE spine-pelvis complex, the intervertebral discs (IVD) consisted of the nucleus pulposus and annulus fibrosus, which included the matrix and the collagen fiber network. A neo-Hookean hyperelastic material model was used to simulate the mechanical behavior of the nucleus pulposus [31], while a hyperelastic fiber-reinforced material model with two families of fibers was used to simulate the mechanical behavior of the annulus [32, 33]. The angulations of the fibers were set to ±30° on a horizontal plane. The values of the material constants used for the IVD in the FE model are listed in Table 2 and are based on the existing literature [33, 34].

Eight groups of important ligaments in the spine-pelvis complex were simulated, i.e., the anterior longitudinal ligament (ALL), the posterior longitudinal ligament (PLL), the sacrotuberous ligament (STL), the sacrospinous ligament (SSL), the anterior sacroiliac ligament (ASL), the posterior sacroiliac ligament (PSL), the sacroiliac interosseous ligament (SIL), and the iliolumbar ligament (ILL). All the ligaments were modeled using the T3D2 element with the tension-only property. Based on the existing literature [34–38], the bilinear elastic material model was used to describe the mechanical behaviors of all the ligaments simulated in the present study (Table 3). The cross-sectional areas of the ligaments are listed in Table 3.

The ligamentous injuries of the pelvis in the pelvic posterior and anterior rings were simulated by removing the connections in the right sacroiliac joint. Two types of posterior pelvic ring injuries were simulated: Tile B and Tile C. Tile B injury (only ligamentous injury) was simulated by removing the anterior sacroiliac ligament, the sacrotuberous ligament, and the sacrospinous ligament on the right side. Tile C injury (only ligamentous injury) was simulated by removing all the ligaments on the right side.

### 2.3. Comparison of the FE Results with Existing Literature

The predictions from the FE model of the intact spine-pelvis complex (i.e., no ligamentous removal and fixations) were compared with in vitro testing data with similar settings (including specimen and loading conditions) [39–42] and other computational results [22]. To make the predictions from the FE model comparable to the in vitro study [39], the same boundary and loading conditions used in [39] were applied in the FE model. Additionally, to ensure that the FE models produce valid results under complex loading scenarios, the results obtained from several loading scenarios were compared. Four loading scenarios were selected and simulated. First, a loading of 294.0 N was applied in the superior-inferior and anterior-posterior directions individually, and then a moment of 42.0 Nm was applied in the flexion and lateral bending directions individually, while both iliac bones were fixed during these loading conditions. At the end of the simulations, the displacements at the center of the sacrum outputted from the FE models under these four loading scenarios were outputted and compared with the existing literature [22, 39–42].

### 2.4. Finite Element Simulation of Different Fixation Methods

**Figure 2:**
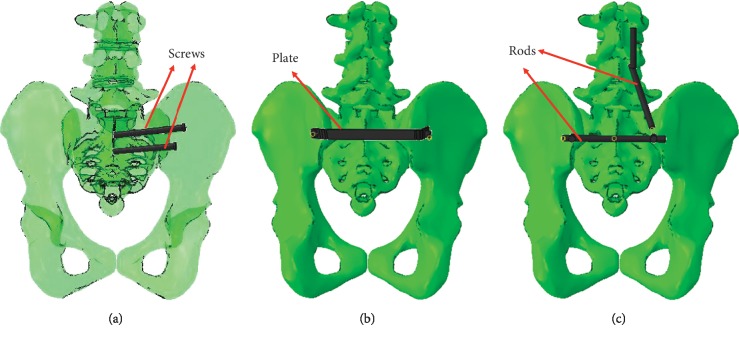
Illustration of different internal fixation methods for posterior pelvic ring injury (a) using screws for the fixation (front view); (b) using a plate for the fixation (back view); and (c) using the modified triangular osteosynthesis (TOS) for the fixation (back view).

The fixation of the ligamentous injury of the anterior ring (ARI) was simulated by connecting the pubic together. Five different methods for fixing the ligamentous injury of the posterior ring (PRI) were compared in the present study:

  Treatment approach 1 (TA1): no fixation for the posterior ring injury, which simulates the conservative treatment for PRI.
  Treatment approach 2 (TA2): use a screw for the fixation of PRI. The screw was inserted from the intersection points between the right anterior superior iliac spine and the posterior superior iliac spine until the tip of the screw reached the first sacral vertebra (S1) (Figure 2(a)). The diameter of the screw is 6.5 mm.
  Treatment approach 3 (TA3): use two screws for the fixation of PRI. The approach for inserting the first screw is the same as that in TA2. The second screw was inserted from the intersection points between the right iliac tubercle and the posterior inferior iliac spine until the tip of the screw reached the second sacral vertebra (S2) (Figure 2(a)). The diameters of both screws are 6.5 mm.
  Treatment approach 4 (TA4): use a plate for the fixation of PRI. The plate was positioned in the plane of the posterior superior iliac spine and fixed using three screws (two on each side of the ilium and one on the posterior side of the ilium) (Figure 2(b)). The width and thickness of the plate are 10.00 mm and 3.00 mm, respectively.
  Treatment approach 5 (TA5) (Figure 2(c)): use the modified triangular osteosynthesis (TOS) for the fixation of the PRI. Two pedicle screws were inserted into the bodies of the third and fourth lumbar vertebrae (L3 and L4). One pedicle screw (Screw Three) was inserted into the right posterior superior iliac spine. Then, a prebent rod was used to connect the three pedicle screws. Another pedicle screw (Screw Four) was also inserted into the right posterior superior iliac spine but was approximately 2.0 cm below Screw Three. In the contralateral position of Screw Four, one pedicle screw (Screw Five) was inserted. Then, a rod was used to connect the Screws Four and Five. The diameters for the pedicle screw and the connecting rod are 6.5 mm and 6.0 mm, respectively.

The plates, bars, and screws used in the fixing are made from titanium alloy. Therefore, a material model with a Young's modulus of 114.0 GPa and a Poisson's ratio of 0.3 was used in the FE models [28]. The threads in the screw were ignored, and the screws were modeled as threadless solids. The plates, bars, and screws are meshed with C3D4 elements. The interfaces between the plates/bars/screws and the bones were modeled with a bonded contact. Based on the fixation status (fixed or nonfixed) of the anterior pelvic ring and the fixation methods for the posterior pelvic ring injury, there are 20 different cases, and consequently, 20 FE models were generated. A mesh-convergent analysis was conducted for each model to ensure no influence of element size on the results. Therefore, each FE model of the complex contained approximately 535,000 elements and 845,000 nodes. For the boundary and loading conditions, a standing posture with a double-leg stance was simulated: the acetabulum on the two sides were fixed in all degrees of freedom, and a vertical force of −500 N was applied on the top surface of the third lumbar vertebral body to simulate the upper body weight (Figure 1(b)). The 20 FE models were solved using Abaqus on a computer workstation (Intel Xeon E-5-2670, 2.60 GHz, 256 GB RAM).

**Figure 3:**
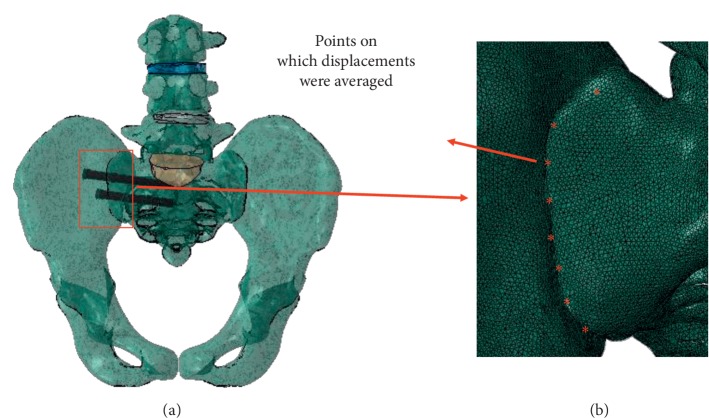
Illustration of the locations where the displacements were averaged over eight points (front view).

For the postprocessing, because the interest of this study was on the deformation/displacement that occurred at the sacroiliac joint, the values of displacements at the sacroiliac joint were calculated to characterize the stability of the pelvis with different fixation approaches. Eight points, located at the outermost boundary of the sacroiliac joint and nearly equally distributed at the boundary, were selected from the sacrum elements, and the average resultant displacement of these points was calculated to quantify the stability of the spine-pelvis complex (Figure 3). First, the resultant displacement at each point was calculated using the following formula:

(1) $$\begin{array}{c} u_{T}=\sqrt{u_{x}^{2}+u_{y}^{2}+u_{z}^{2}}, \end{array}$$

where *u*_*x*_, *u*_*y*_, and *u*_*z*_ are the displacements in the *x*-, *y*- and *z*-axis, respectively. Then, the average resultant displacement was calculated as the average value of the eight resultant displacements.

## 3. Results

### 3.1. Comparison of the FE Predictions with Existing Literature

**Figure 4:**
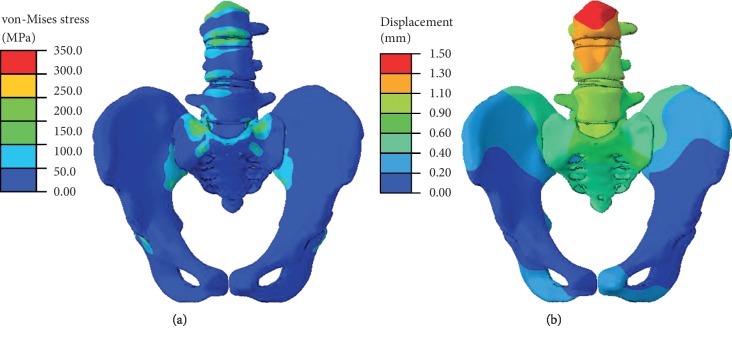
Distributions of the von-Mises stress (a) and displacement (b) in the FE model of the spine-pelvis complex (front view).

A comparison of the predictions from the FE model of the intact spine-pelvis system with published in vitro test data is presented in Table 4. The displacements at the center of the sacrum predicted from the present FE model are 0.32 mm under the superior-inferior loading, 0.55 mm under the anterior-posterior loading, 1.23 mm under the flexion loading, and 0.41 mm under the lateral bending loading, which agree well with the in vitro data, i.e., 0.28 ± 0.25 mm (superior), 0.48 ± 0.38 mm (anterior), 1.31 ± 0.60 mm (flexion), and 0.37 ± 0.27 mm (lateral bending) [39]. Additionally, the magnitude of the displacement predicted from the present FE model is in good agreement with other in vitro studies (Table 4). Furthermore, the displacement predicted from the present FE model is similar to that predicted from the FE pelvis model developed by Zhang et al. [22], i.e., 0.32 mm (the present study) vs. 0.38 mm (Zhang et al. [22]) under the superior-inferior loading case. The distributions of the von Mises stress and displacement predicted from the present intact FE spine-pelvis model are presented in Figure 4.

### 3.2. Comparison of the Performance of Different Fixation Methods

The spine-pelvis complex fixed with treatment approach 5 (the modified TOS method) has the best stability. The resultant displacements at the sacroiliac joint are the smallest (1.5 mm, 1.6 mm, 1.6 mm, and 1.7 mm) (Table 5) compared with other treatments in all four injury cases. The complex fixed with treatment approach 3 (using two screws for fixation) has the second best stability. The resultant displacements at the sacroiliac joint for different injury cases are 1.6 mm, 1.7 mm, 1.7 mm, and 1.8 mm, and compared with TA5, the resultant displacements are increased by 6.7%, 6.3%, 6.3%, and 5.9%. The complex fixed with treatment approach 2 (using one screw for fixation) has the third best stability. The resultant displacements at the sacroiliac joint for different injury cases are 1.8 mm, 1.9 mm, 2.0 mm, and 2.1 mm, and compared with TA5, the resultant displacements are increased by 20.0%, 18.7%, 25.0%, and 23.5%. The resultant displacements at the sacroiliac joint treated with approach 4 (using a plate for fixation) are 2.3 mm, 2.4 mm, 4.8 mm, and 4.9 mm for different injury cases, and compared with TA5, the resultant displacements are increased by 53.3%, 50.0%, 200.0%, and 188.2%. Treatment approach 1 (conservative method) has the worst stability, with resultant displacements of 3.1 mm, 3.2 mm, 6.3 mm, and 6.5 mm.

### 3.3. Comparison of the Stability of the Complex between the Anterior Pelvic Ring Fixed and Nonfixed Models and between Tile B and Tile C Injury Cases

The fixation of the anterior pelvic ring only slightly increased the stability of the spine-pelvis complex. Under the situation of Tile B posterior ring injury and anterior ring injury fixed, the resultant displacements at the sacroiliac joint are 3.1 mm, 1.8 mm, 1.6 mm, 2.3 mm, and 1.5 mm for the fixation approaches of TA1, TA2, TA3, TA4, and TA5, respectively (Table 5). Under the situation of Tile B posterior ring injury, compared with the anterior ring injury nonfixed case (R2), the resultant displacements at the sacroiliac joint for the anterior ring injury fixed case (R1) were reduced by 3.1%, 5.3%, 5.9%, 4.2%, and 6.3%, respectively (Table 6). Under the situation of Tile C posterior pelvic ring injury, the same trend was found: resultant displacements at the sacroiliac joint were 6.3 mm, 2.0 mm, 1.7 mm, 4.8 mm, and 1.6 mm for different fixation approaches in the case of anterior ring injury fixed; in the anterior ring fracture nonfixed case, the resultant displacements were reduced by 3.0%, 4.8%, 5.6%, 2.1%, and 5.9%, respectively (Table 6).

For fixing Tile B and Tile C posterior ring injuries, different fixation approaches have different performances. TA1 and TA4 have similar performances; compared with Tile C, the resultant displacements were decreased by 50.8% and 52.1% for the anterior ring injury fixed case and decreased by 50.7% and 51.0% for the anterior ring injury nonfixed case (Table 6). TA2, TA3, and TA5 have similar performances; compared with Tile C, the resultant displacements were decreased by 10.0%, 5.6%, and 6.3% for the anterior ring injury fixed case and by 9.5%, 5.5%, and 5.9% for the anterior ring injury nonfixed case (Table 6).

## 4. Discussion

In the present study, the biomechanical performances of five different internal methods for fixing posterior pelvic ring injury were analyzed using the finite element method.

Regarding the modified triangular osteosynthesis (TOS) method, it is revealed in the present study that the performance of this approach is the best for fixing a posterior pelvic ring injury. However, it should be noted that this finding was made based solely on the static linear displacement analysis of the system, and an analysis of screw loosening and failure behavior was not considered. Considering that in vertical shear injuries, a higher frequency of loosened sacroiliac screws was reported [43], the present study should be extended in the future to provide a comprehensive understanding of the performance of TOS. It should also be noted that in the traditional TOS method, the pedicle screws are fixed between L4 and L5 or between L5 and S1, and the connecting rod needs to be prebent into an “S” shape, which may largely reduce its strength. In addition, in clinical practice, the paravertebral muscles may need to be cut away to insert the connecting rod. Therefore, the traditional TOS approach is time-consuming, and large traumas may be induced by this operation. In the modified TOS fixation method, L3 and L4 are fixed using pedicle screws. In this case, the prebending of the connecting rod becomes easier and its strength will be maintained. Previous reports have shown that there are no cases of broken screws in the clinic after the application of the TOS method [23, 44]. It is shown in the present study that the modified TOS fixation method performs very well in fixing posterior pelvic ring injuries for different injury cases. In the clinical setting, this technique is widely used for Tile C injury, but because of the large trauma induced, for Tile B injury, other fixation approaches are recommended for fixing posterior pelvic ring injuries after the fixation of the anterior pelvic ring.

Regarding the TA2 (using one screw located towards S1) and TA3 (using two screws located towards S1 and S2) approaches, it is shown in the present study that these two approaches have similar performance in fixing a posterior pelvic ring injury. In most cases, one screw is enough to fix the posterior pelvic ring. However, for Tile C injury, there is a large shear force at the posterior pelvic ring, and consequently, bending and injury of the screw can easily occur in the clinic. Therefore, it is necessary to insert the second screw to prevent compromising the screw. Previous studies have also shown the good performance of TA2 and TA3 in fixing injuries in the sacrum [45]. It should be noted that the screws can be inserted in a minimally invasive manner, and thus minimal trauma is induced. Furthermore, the screws can be accurately positioned with the help of medical imaging techniques [46].

Regarding the approach of TA4 (using a plate positioned at the posterior superior iliac spine), it is revealed in the present study that this technique is not as effective as other fixation approaches. The possible reason for this is that the plate is fixed on the two ends of the ilium and not directly on the sacrum. When the sacroiliac joint is injured, the fixing plate only prevents the separation of the two ends of the ilium and has only a very limited effect on the stability of the posterior pelvic ring. It should be noted that the TA4 procedure has the advantages of a small incision, fewer complications, and a short time in the hospital [47]. In the cli nic, TA4 is suitable for Tile B pelvic injury but is not recommended for Tile C pelvic injury.

Another important finding from the present study is that the fixation of the anterior pelvic ring injury only slightly increased the stability of the spine-pelvis complex. The relative percentage differences in the resultant displacement between the anterior ring injury fixed and nonfixed models are in the range of 1.4%–5.9% (lower values in the fixed models) for different fixation approaches (Table 5). When comparing the performances of different fixation approaches for fixing Tile B and Tile C posterior ring injuries, it was found that the resultant displacements were relatively largely changed (in the range of 50.7% to 52.1%) when TA1 and TA4 were applied and only slightly changed (in the range of 5.5% to 10.0%) when TA2, TA3, and TA5 were applied (Table 5). The reason is that the motions constrained by the ligaments removed in the case of Tile C were partially reconstrained by the implants of TA2, TA3, and TA5, while TA1 and TA4 did not replace the role of ligaments removed in Tile C injury.

Because of the difficulties in the mechanical testing of human cadavers, e.g., large intersubject variances and infeasibilities in measuring stresses inside the implants and bones, finite element analysis (FEA) has become an important tool in assessing the performance of implants [48–51]. Additionally, it is a major challenge to compare intact, injured, and treated situations using cadaveric specimens because of the large variations caused by bone anatomy, bone density, and fracture patterns [52, 53]. The FEA approach has the feature of being able to simulate different scenarios on the same sample and thus has been widely used in comparison studies [48]. However, because different FE model setups (loading, boundary condition, implant design, and so on.) were used in different studies [48, 54], a direct quantitative comparison of the results from the present study with those in the literature is not possible. Qualitatively, the conclusion obtained from the present study agrees well with those in the literature [23, 44, 55], i.e., modified triangular osteosynthesis is an effective way to fix posterior pelvic ring fractures.

Nevertheless, some potential limitations related to the present FE models should be noted. First, a simplified FE spine-pelvis model was used in the present study. Regarding the numerical modeling of the mechanical behavior of bone tissues, an isotropic linear elastic material model was used, although bone tissues are intrinsically nonlinear, anisotropic, viscoelastic, and heterogeneous [56]. However, bone tissues are brittle, and at the elastic deformation stage, nearly linear behavior was found for the bone tissues [57, 58]. The anisotropic property is mainly for describing the behavior of porous cancellous bone tissues, while for the dense cortex and endplates, their mechanical behaviors can well be simplified as isotropic [57]. In the present study, the main part of interest and the part transferring the loading is the dense cortex. The viscoelastic properties of a material (e.g., creep, rate dependent, and relaxation) mainly describe the long-term effect/behavior of the material [59], while in the present study, only the static, short-term behaviors of the spine-implant system are investigated. Considering all these facts, the application of an isotropic linear elastic model for describing the mechanical behaviors of bone tissues is appropriate and valid in the present study. Regarding the numerical modeling of the mechanical behavior of ligaments, bilinear elastic material models were used because the loading applied in the present study is small, and the initial stage of ligament behavior can be well simulated using a bilinear elastic model [18, 30]. Furthermore, it should be noted that the skeletal muscles around the pelvis are not reconstructed, and the authors are in the process of reconstructing them for use in a model to investigate the role of skeletal muscles in the stability of the spine-pelvis complex [60]. Second, the long-term stability of the spine-implant system (e.g., fatigue and implant loosening due to tissue adaptation) was not investigated in the present study. It should be noted that the investigation of the long-term stability of a spine-implant system requires the definition of complex material models for human tissues in FE models, which is still under the development stage in the biomechanics community at the moment. Considering these facts, only static FE analysis, which is reliable and widely accepted in the biomechanics community, was performed in the present study. Third, only one loading scenario (upright standing) was simulated in the present study. Standing is the most common posture in daily activities, and thus, the results from this scenario are of high value for both surgeons and patients. However, other loading scenarios, such as lateral bending and forward bending [61], should be simulated to provide a comprehensive analysis of the performance of different internal fixation methods. Finally, only one FE model was generated to evaluate the performance of different fixation methods in the present study. The influences of the variability among different human subjects (e.g., the bone properties and anatomical differences) and the FE modeling uncertainties (e.g., the bone density-modulus relationship) on the results are not investigated [62, 63]. However, the main factors influencing the stability of the spine-implant system are the type of fixation method and the type of fixation screws and plates. Compared with the influence of these main factors, the influence of the variability among human subjects on the results might be small. Therefore, it is believed that the conclusion made in the present study may not be changed if more case studies covering the variabilities among subjects are performed. However, in the future, an investigation of the influences of the variabilities among subjects and uncertainties in the FE models should still be performed using advanced numerical modeling techniques, such as stochastic modeling and principal component analysis [62–64].

## 5. Conclusions

In summary, it was found in the present study that the best internal fixation method for fixing posterior pelvic ring injury is the modified triangular osteosynthesis approach, followed by S1 + S2 screw fixation, S1 screw fixation, and plate fixation. This study provides guidance on the selection of fixation methods for posterior pelvic ring injury.

## Figures and Tables

**Table 1:** Material properties and element types for the linear elastic structures in the FE model.

Component	Young's modulus (MPa)	Poisson's ratio	Element type	Reference
Cortical shell	12,000	0.3	C3D4	Burstein et al. [24]
Cancellous bone	1500	0.3	C3D4	Lindahl [25]
Endplate	12,000	0.3	C3D4	Grant et al. [26]
Posterior structure	3500	0.3	C3D4	Shirazi-Adl et al. [27]
Plates/bars/screws (titanium alloy)	114,000.00	0.3	C3D4	Xu et al. [28]

**Table 2:** Material constants for the intervertebral disc.

Component	Material constants	Element type	Reference
Nucleus pulposus	*C*_10_ = 0.16 MPa *D* = 0.024 MPa^−1^	C3D4	Kasra et al. [35]; Schirazi-Adl et al.[36]

Annulus matrix	*C*_10_ = 0.1 MPa *C*_20_ = 2.5 MPa *D* = 0.306 MPa^−1^	C3D4	Moramarco et al. [34]; Eberlein et al. [33]
Annulus fibers	*K*_1_=1.8 [MPa] *K*_2_=11.0*α*=±30°		

**Table 3:** Material properties, element types, and cross-sectional area of ligaments in the FE model.

Ligaments	Strain (%)	Stiffness (N/mm)	Strain (%)	Stiffness (N/mm)	Area (mm^2^)	Reference
Anterior longitudinal ligament (ALL)	0 < *ε* < 12.2	347	*ε* ≥ 12.2	787	32.4	Moramarco et al. [34] Rohlmann et al. [38] Naserkhaki et al. [37]
Posterior longitudinal ligament (PLL)	0 < *ε* < 11.2	295	*ε* ≥ 11.2	617	5.2	
Sacrotuberous ligament (STL)	0 < *ε* < 18.2	467	*ε* ≥ 18.2	988	42.85	
Sacrospinous ligament (SSL)	0 < *ε* < 13.9	387	*ε* ≥ 13.9	897	35.6	
Anterior sacroiliac ligament (ASL)	0 < *ε* < 22.1	454	*ε* ≥ 22.1	887	112	
Posterior sacroiliac ligament (PSL)	0 < *ε* < 21.8	398	*ε* ≥ 21.8	876	96	
Sacroiliac interosseous ligament (SIL)	0 < *ε* < 17.2	412	*ε* ≥ 17.2	932	39	
Iliolumbar ligament (ILL)	0 < *ε* < 18.5	432	*ε* ≥ 18.5	957	21	

**Table 4:** Comparison of the FE predictions with the in vitro test data.

Test set-up	Biomechanical findings	References
Both iliac bones fixed, 294.0 N in superior-inferior and anterior-posterior directions, and 42 Nm moment in the flexion and lateral bending directions	Displacements at the center of the sacrum are around 0.32 mm (superior), 0.55 mm (anterior), 1.23 mm (flexion), and 0.41 mm (lateral bending)	The present study

294.0 N applied in the superior, inferior, anterior, posterior, and lateral directions of the sacroiliac joints	The mean displacements are around 0.28 mm (superior), 0.48 mm (anterior), 1.31 mm (flexion), and 0.37 mm (lateral bending)	Miller et al. [39]

Bilateral stance simulated using the intact sacroiliac joint and public symphysis	The mean displacement in the intact pelvis is around 0.2 mm in the stance posture	Varga et al. [40]

Quasistatic compressive loading applied in the pelvis	After fixation, the displacement magnitudes at the fracture sites were small (mean = 0.09 mm)	Sawaguchi et al. [41]

Cyclic increasing axial loading applied through the acetabulum	The mean displacements at the screw and plates are around 0.37 mm and 0.11 mm after 100 cyclic loading	Acklin et al. [42]

**Table 5:** Comparison of the average resultant displacement at the sacroiliac joint for different fixation methods.

	TA1 (mm)^*∗*^	TA2 (mm)	TA3 (mm)	TA4 (mm)	TA5 (mm)
Tile B-ARI (R1)^*∗∗*^	3.1	1.8	1.6	2.3	1.5
Tile B-ARNF (R2)^*∗∗*^	3.2	1.9	1.7	2.4	1.6
Tile C-ARI (R3)	6.3	2.0	1.7	4.8	1.6
Tile C-ARNF (R4)	6.5	2.1	1.8	4.9	1.7

^*∗*^“TA1” represents treatment approach 1 for posterior pelvic ring injury, and so on; ^*∗∗*^“ARI” and “ARNF” represent the fixation and nonfixation of the anterior pelvic ring and so on.

**Table 6:** Relative percentage difference between different fixation methods under different cases of pelvic ring fractures.

	TA1^*∗*^ (%)	TA2 (%)	TA3 (%)	TA4 (%)	TA5 (%)
(R1^*∗∗*^ − R2)/R2	−3.1	−5.3	−5.9	−4.2	−6.3
(R3 − R4)/R4	−3.0	−4.8	−5.6	−2.1	−5.9
(R1 − R3)/R3	−50.8	−10.0	−5.6	−52.1	−6.3
(R2 − R4)/R4	−50.7	−9.5	−5.5	−51.0	−5.9

^*∗*^“TA1” represents treatment approach 1 for posterior pelvic ring injury and so on; ^*∗∗*^“R1” represents the case of Tile B anterior pelvic ring fixation, as shown in Table 5, and so on.

## Data Availability

The datasets used and/or analyzed during the current study are available from the corresponding author on reasonable request.
